# Glycolysis is the primary bioenergetic pathway for cell motility and cytoskeletal remodeling in human prostate and breast cancer cells

**DOI:** 10.18632/oncotarget.2766

**Published:** 2014-11-16

**Authors:** Takumi Shiraishi, James E. Verdone, Jessie Huang, Ulf D. Kahlert, James R. Hernandez, Gonzalo Torga, Jelani C. Zarif, Tamir Epstein, Robert Gatenby, Annemarie McCartney, Jennifer H. Elisseeff, Steven M. Mooney, Steven S. An, Kenneth J. Pienta

**Affiliations:** ^1^ Department of Urology, The James Buchanan Brady Urological Institute, Johns Hopkins University, Baltimore, MD, USA; ^2^ Department of Oncology, Johns Hopkins University, Baltimore, MD, USA; ^3^ Department of Pharmacology and Molecular Sciences, Johns Hopkins University, Baltimore, MD, USA; ^4^ Department of Chemical and Biomolecular Engineering, Johns Hopkins University, Baltimore, MD, USA; ^5^ Department of Environmental Health Sciences, Johns Hopkins Bloomberg School of Public Health, Baltimore, MD, USA; ^6^ Department of Pathology, Division of Neuropathology, The Johns Hopkins Hospital, Baltimore, MD, USA; ^7^ Department of Integrative Mathematical Oncology and Department of Diagnostic Imaging, H. Lee Moffitt Cancer Center & Research Institute, Tampa, FL, USA; ^8^ The Wilmer Eye Institute and Department of Biomedical Engineering, Baltimore, MD, USA; ^9^*In Vivo* Cellular and Molecular Imaging Center, The Johns Hopkins School of Medicine, Baltimore, MD, USA

**Keywords:** cytoskeleton, motility, cancer metabolism, glycolysis, metastasis

## Abstract

The ability of a cancer cell to detach from the primary tumor and move to distant sites is fundamental to a lethal cancer phenotype. Metabolic transformations are associated with highly motile aggressive cellular phenotypes in tumor progression. Here, we report that cancer cell motility requires increased utilization of the glycolytic pathway. Mesenchymal cancer cells exhibited higher aerobic glycolysis compared to epithelial cancer cells while no significant change was observed in mitochondrial ATP production rate. Higher glycolysis was associated with increased rates of cytoskeletal remodeling, greater cell traction forces and faster cell migration, all of which were blocked by inhibition of glycolysis, but not by inhibition of mitochondrial ATP synthesis. Thus, our results demonstrate that cancer cell motility and cytoskeleton rearrangement is energetically dependent on aerobic glycolysis and not oxidative phosphorylation. Mitochondrial derived ATP is insufficient to compensate for inhibition of the glycolytic pathway with regard to cellular motility and CSK rearrangement, implying that localization of ATP derived from glycolytic enzymes near sites of active CSK rearrangement is more important for cell motility than total cellular ATP production rate. These results extend our understanding of cancer cell metabolism, potentially providing a target metabolic pathway associated with aggressive disease.

## INTRODUCTION

Tumor metastasis is responsible for more than 90% of cancer-related deaths [1]. Multiple steps are involved in the process of metastasis, classically including local invasion, intravasation, transport, extravasation, and colonization [2, 3]. The successful dissociation of a cancer cell from the primary tumor to a distant metastatic site requires a high degree of cell motility associated with an aggressive cancer phenotype and is often associated with expression of mesenchymal biomarkers [4-6]. The motility of different cancers has been studied extensively and cell motility is driven by actin-dependent cytoskeletal reorganization [7, 8].

Glycolysis and oxidative phosphorylation are the two major energy producing pathways in the cell. Dynamic shifts between these two processes are observed in most cells as they adapt to environmental changes for the purpose of survival. The balance between these two processes may contribute to the determination of specific lineage decisions [9-12] and stem cell development [13, 14]. In cancer, most cells exhibit increased glycolysis and use this metabolic pathway for generation of ATP even in the presence of O_2_ [15]. Potential reasons for aerobic glycolysis in cancer have been explained by malfunction of mitochondria, adaptation to a hypoxic environment, oncogenic signals, altered metabolic enzymes [16] and a physiological response to enhanced energy demand for membrane transporter activity required for cell division, growth and migration [17]. Accumulating evidence suggests that the high rate of glycolysis in tumors not only compensates for increased ATP demand, but also contributes to cell proliferation and survival by affecting signaling pathways and enhancing the production of macromolecules such as proteins, nucleic acids, and lipids [18, 19]. Since glycolytic shift is associated with a more aggressive phenotype [20], it has also been suggested that aerobic glycolysis is a hallmark for invasive cancers [21]. Previous studies of tumor cell metabolism suggest increased cancer cell utilization of the glycolytic pathway contributes to cell chemotaxis, a process required for metastasis [22]. The extent to which aerobic glycolysis relates to tumor metastasis is not fully understood.

The link between carbohydrate metabolism, glycolytic enzyme localization, and regulation of the cytoskeleton has been studied extensively [23-27]. To further understand metabolic alterations in cancer progression and their impact on cellular motility and actin-dependent cytoskeletal dynamics, in the present study, we evaluated bioenergetic profiles of epithelial and mesenchymal cancer cell models with respect to proton production rate (PPR) and oxygen consumption rate (OCR). We further examined the association of these measures with cell motility and cytoskeletal (CSK) remodeling. Our results demonstrate that cell motility and cytoskeleton remodeling are dependent on aerobic glycolysis but not oxidative phosphorylation, suggesting that ATP localization with sites of active cytoskeletal remodeling rather than total cellular ATP content is necessary for cancer cell movement. This work provides a new framework for understanding the energy and metabolism requirements of aggressive and less-aggressive cancer phenotypes.

## RESULTS

### Aggressive mesenchymal cancer cells exhibit distinct cytoskeletal dynamics from those of less-aggressive epithelial cancer cells

**Figure 1:**
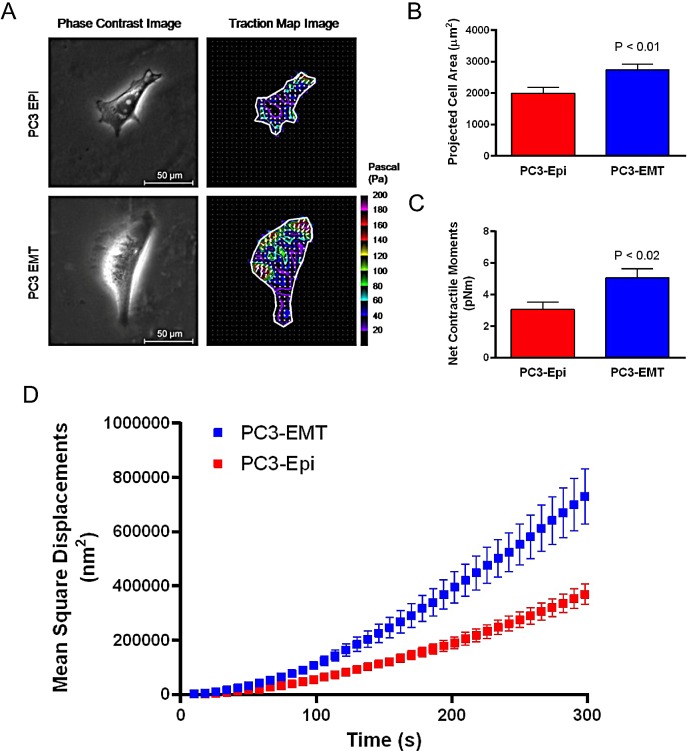
PC3-EMT cells are biophysically distinct from PC3-Epi cells (A) PC3-EMT and PC3-Epi cells were plated on polyacrylamide gels, and phase contrast and traction map images of representative cells are shown. Through FTTM, the (B) projected cell area and (C) net contractile moments were obtained. Data are represented as mean ± SE (*n* = 12 for PC3-EMT, *n* = 10 for PC3-Epi). (D) Remodeling represented by mean square displacements obtained from the spontaneous nanoscale bead motion in PC3-Epi and PC3-EMT cells. FTTM: Fourier transform traction microscopy.

In order to understand the biophysical features associated with epithelial (PC3-Epi) and mesenchymal (PC3-EMT) prostate cancer cells, we measured cell traction forces with Fourier transform traction microscopy and CSK remodeling dynamics by *spontaneous* motions of beads functionalized to the living CSK through cell surface integrin receptors [28]. Compared to PC3-Epi cells, PC3-EMT cells spread to a larger size and exerted greater cell traction forces (Figures 1A-1C). The net contractile moment, which provides a scalar measure of the cell's contractile strength, was approximately 1.7-fold higher (P<0.02) in PC3-EMT cells compared to PC3-Epi cells (Figure 1C). PC3-EMT cells also displayed faster CSK remodeling dynamics than PC3-Epi cells (Figure 1D). These results indicate that mesenchymal PC3-EMT cells exhibit distinct cytoskeletal dynamics from epithelial PC3-Epi cells.

### Mesenchymal cancer cells exhibit a high rate of aerobic glycolysis

**Figure 2:**
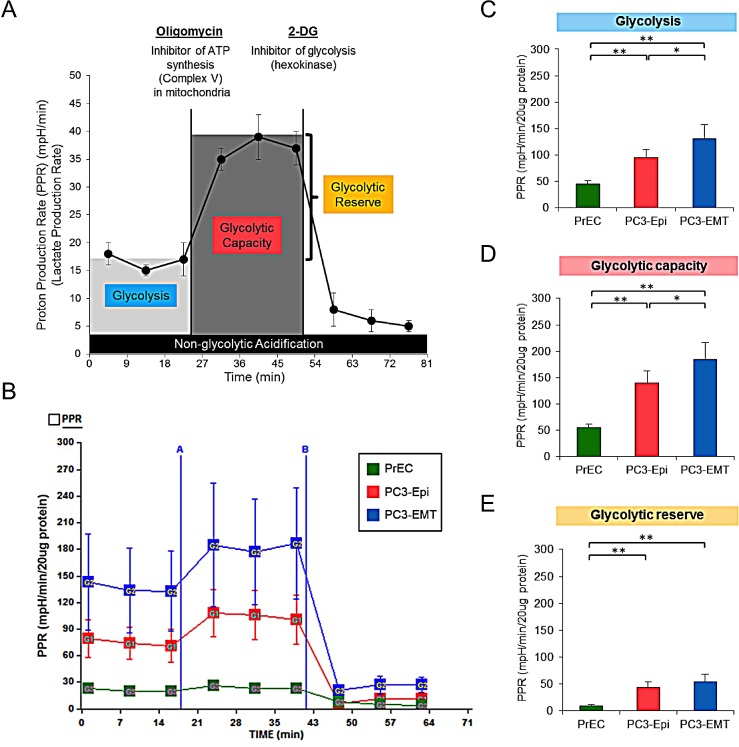
PC3-EMT cells have higher glycolytic activity compared to PC3-Epi cells (A) Example of proton production rate (PPR) analyzed by a Seahorse Bioscience XF24 Extracellular Flux Analyzer when oligomycin and 2-deoxy-D-glucose (2-DG) were injected. Glycolysis, glycolytic capacity and glycolytic reserve were calculated as shown in the image. (B) Representative traces of PPR in PC3-Epi, PC3-EMT and PrECs. PPR was measured continuously throughout the experimental period at baseline followed by the addition of the indicated drugs. A; oligomycin (1uM), B; 2-DG (100mM). Glycolysis (C), glycolytic capacity (D) and glycolytic reserve (E) were calculated from the mean of three baseline readings. The independent biological experiments were repeated at least three times. Data were represented as the mean ± SD from 6 or 7 Seahorse microplate wells. *P<0.05, **P<0.01.

We next examined glycolytic activity of PC3-Epi, PC3-EMT and non-cancer prostate epithelial cells (PrECs) by measuring proton production rate (PPR), which is associated with the production of lactic acid (Figure 2A). Under basal condition, glycolytic activity (glycolysis) was highest in PC3-EMT cells, followed by PC3-Epi and PrECs (Figures 2B and 2C). Oligomycin was then added to inhibit mitochondrial ATP synthesis followed by 2-deoxy-D-glucose (2-DG), a non-competitive inhibitor of hexokinase that blocks glycolysis (Figure 2A). This experimental design provides an estimation of glycolytic capacity and glycolytic reserve under mitochondrial dysfunction (Figure 2A). The highest glycolytic capacity and glycolytic reserve were observed in PC3-EMT cells in the presence of oligomycin (Figures 2B, 2D, and 2E). In order to confirm the results that mesenchymal cancer cells exhibited higher glycolysis compared to epithelial cancer cells, PPR was also examined using another epithelial and mesenchymal cancer cell models derived from breast cancer cell lines. In this experiment, we used parental mesenchymal MDA-MB-231 cells (MDA-EMT) and MDA-MB-231 cells that stably overexpress the epithelial inducing transcription factors OVO-like 1 and OVO-like 2 (MDA-Epi) [29]. Consistent with the data obtained from PC3-Epi and PC3-EMT cells, MDA-EMT cells exhibited higher glycolysis compared to MDA-Epi cells (Figure S1). Altogether, these results suggest that mesenchymal cancer cells exhibit a higher rate of aerobic glycolysis than epithelial cancer cells.

### ATP synthesis from oxidative phosphorylation in epithelial and mesenchymal cancer cells is equivalent

**Figure 3:**
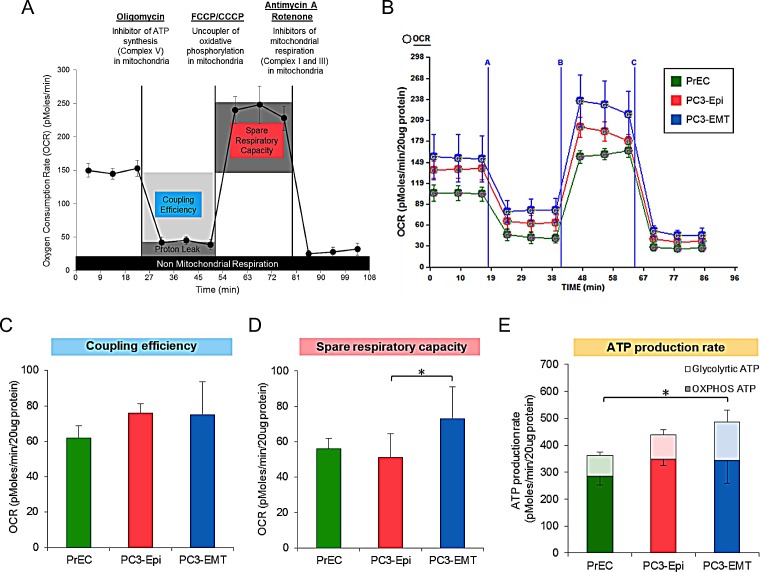
PC3-Epi and PC3-EMT cells exhibit a similar mitochondrial ATP production rate (A) Example of oxygen consumption rate (OCR) analyzed by a Seahorse Bioscience XF24 Extracellular Flux Analyzer when oligomycin, carbonyl cyanide-4-(trifluoromethoxy)phenylhydrazone or carbonyl cyanide meta-chlorophenyl hydrazone (FCCP/CCCP, respectively), and Antimycin A with Rotenone were injected. Coupling efficiency, spare respiratory capacity and non-mitochondrial respiration were calculated as shown in the image. (B) Representative traces of OCR in PC3-Epi, PC3-EMT and PrECs. OCR was measured continuously throughout the experimental period at baseline followed by the addition of the indicated drugs. A; oligomycin (1uM), B; FCCP (200nM for PC3-Epi and PC3-EMT cells, 400nM for PrECs), C; Antimycin A (2uM) and Rotenone (2uM). Coupling efficiency (C) and spare respiratory capacity (D) were calculated from the mean of three baseline readings. (E) ATP production rate was calculated from OCR and PPR measured in the Seahorse Bioscience XF24 Extracellular Flux Analyzer by the following equation; ATP production rate = OCR × 4.6 + PPR x 1. The independent biological experiments were repeated at least three times. Data were represented as the mean ± SD from 6 or 7 Seahorse microplate wells. *P<0.05.

Since it has been reported that mitochondrial activity is associated with specific lineage decision, oxygen consumption rate (OCR) as a measure of mitochondrial respiration in PC3-Epi, PC3-EMT and PrECs was then determined (Figure 3A). No significant difference was observed in the coupling efficiency (the total basal OCR minus the OCR after the addition of oligomycin) (Figures 3B and 3C). Subsequently, the uncoupling agent, carbonyl cyanide-4-(trifluoromethoxy)phenylhydrazone (FCCP), was administered to examine the maximal turnover of the electron transport chain uncoupled from ATP synthesis (spare respiratory capacity). The spare respiratory capacity in PC3-EMT cells was significantly higher than PrECs and PC3-Epi cells (Figures 3B and 3D), indicating that PC3-EMT cells are more adaptable to stress. Furthermore, total ATP production rate was significantly higher in PC3-EMT cells than in PrECs (Figure 3E). Although the total ATP production rate in PC3-EMT cells tended to be higher compared to PC3-Epi cells, the differences were not significant (Figure 3E). The higher glycolytic ATP contributed to the increase of total ATP production rate in PC3-EMT cells compared to PC3-Epi cells (Figure 3E). Consistent with the data obtained from PC3-Epi and PC3-EMT cells, no significant difference was observed in coupling efficiency and ATP production rate between MDA-Epi and MDA-EMT cells (Figures S2A,2B, and S2D). Furthermore, the spare respiratory capacity in MDA-EMT cells was significantly higher than MDA-Epi cells (Figure S2A and S2C), further supporting the concept that mesenchymal cancer cells have an increased ability to adapt to stressful conditions. Taken together, these results indicate that mesenchymal cancer cells possess higher glycolytic activity compared to epithelial cancer cells without a difference in mitochondrial respiration.

### Expression and activity of glycolytic enzymes are up-regulated in mesenchymal cancer cells

**Figure 4:**
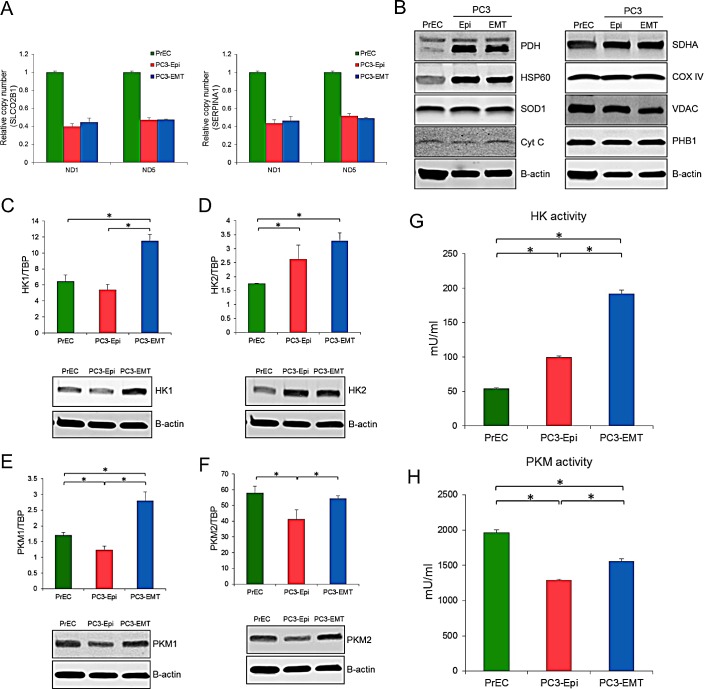
Higher expression and activity of glycolytic enzyme in PC3-EMT cells The ratio of NADH dehydrogenase subunit 1 (ND1) and NADH dehydrogenase subunit 5 (ND5) to SLCO2B1 (A) and SERPINA1 (B) DNA in PrECs, PC3-Epi and PC3-EMT cells. The amount of ND1, ND5, SLCO2B1 and SERPINA1 were analyzed by Q-PCR. Data were represented as the mean ± SD from three independent experiments. (C) Mitochondrial proteins were detected by western blots in PrECs, PC3-Epi, and PC3-EMT cells. Β-actin was used as a loading control. HK1 (C), HK2 (D), PKM1 (E) and PKM2 (F) mRNA expression (upper) were determined in PrECs, PC3-Epi and PC3-EMT cells by Q-PCR. The expression of mRNA was normalized to TATA binding protein (TBP). Data were represented as the mean ± SD from three independent experiments. Western blots for HK1 (C), HK2 (D), PKM1 (E) and PKM2 (F) protein expression (lower) in PrECs, PC3-Epi and PC3-EMT cells. Β-actin was served as a loading control. Hexokinase (G) and pyruvate kinase (H) activities in PrECs, PC3-Epi, and PC3-EMT cells. The independent biological experiments were repeated at least three times. Data were represented as the mean of triplicate experiments ± SD. *P<0.05.

To confirm the same OCR levels devoted to ATP synthesis in epithelial and mesenchymal cancer cells, various parameters of mitochondrial content were analyzed. The ratio of mitochondrial DNA (mtDNA) to nuclear DNA was the same between PC3-Epi and PC3-EMT cells (Figure 4A). The high ratio of mtDNA to nuclear DNA in PrECs likely reflects the aneuploidy of PC3 cells since the modal chromosome number of PC3 cells are 62 [30]. Analysis of several proteins associated with mitochondrial function revealed no significant difference in PC3-Epi and PC3-EMT cells, although several proteins including pyruvate dehydrogenase (PDH), heat shock protein 60 (HSP60), and succinate dehydrogenase complex, subunit A (SDHA) were all up-regulated in PC3-Epi and PC3-EMT cells relative to PrECs (Figure 4B). MDA-Epi and MDA-EMT cells also possess the similar levels of mitochondrial contents with regard to the ratio of mtDNA to nuclear DNA and several mitochondrial proteins (Figures S3A and S3B). The expression and activity of several key enzymes involved in the glycolytic pathway including hexokinase 1 (HK1), hexokinase 2 (HK2), M1 isoform of pyruvate kinase (PKM1), and M2 isoform of pyruvate kinase (PKM2) were next examined. All enzymes except for HK2 were significantly up regulated at both mRNA and protein levels in PC3-EMT cells when compared to PC3-Epi cells (Figures 4C-4F). Furthermore, the enzymatic activity of HK and PKM was significantly higher in PC3-EMT cells than in PC3-Epi cells (Figures 4G and 4H). PrECs exhibited the highest PKM activity as well as PKM2 expression among those cells. MDA-EMT cells also exhibited higher PKM activity than MDA-Epi cells, although HK activity was the same between MDA-Epi and MDA-EMT cells (Figure S3G and S3H). Taken together, these findings further support the idea that mesenchymal cancer cells have a higher rate of aerobic glycolysis without any change in mitochondrial ATP synthesis when compared to epithelial cancer cells.

### Inhibition of glycolysis, but not mitochondrial ATP synthesis, attenuates cell motility

**Figure 5:**
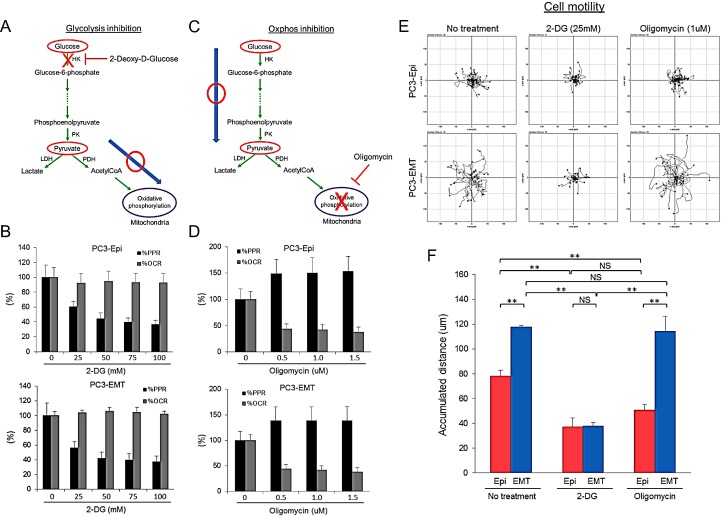
Inhibition of glycolysis, but not mitochondrial ATP synthesis, attenuates cell motility (A) Schema of the effect of 2-deoxy-D-glucose (2-DG) in the presence of pyruvate on glycolytic pathway and TCA cycle. 2-DG treatment in the presence of pyruvate blocks glycolysis without affecting mitochondrial respiration. (B) Effect of 2-DG on PPR and OCR in PC3-Epi (upper) and PC3-EMT (lower) cells. Data are represented as percent change of PPR and OCR compared to base line. (C) Schema of the effect of oligomycin on glycolytic pathway and TCA cycle. Oligomycin treatment blocks ATP synthesis in mitochondria while glycolytic pathway is not affected. (D) Effect of oligomycin on PPR and OCR in PC3-Epi (upper) and PC3-EMT (lower) cells. Data are represented as percent change of PPR and OCR compared to base line. PC3-Epi and PC3-EMT cells were cultured in XF minimal basal medium supplemented with 11mM glucose, 1mM sodium pyruvate and 1×GlutaMax (Invitrogen) in the presence or absence of 2-DG (25mM) or oligomycin (1uM). (E) Representative tracks of cell movements that were traced and visualized using Manual Tracking and Chemotaxis tool of ImageJ software every 15 min for 6 hrs. (n=38 and 25 for control, n=31 and 29 for 2-DG treatment and n=45 and 29 for oligomycin treatment in PC3-Epi and PC3-EMT cells, respectively.) (F) The accumulated distance analyzed by chemotaxis tool of ImageJ software. Data are represented as mean of triplicate experiments ± SD. **P<0.01. NS; not significant.

Mesenchymal cancer cells exhibited distinct biophysical features as well as a higher rate of aerobic glycolysis as compared to epithelial cancer cells. To investigate the impact of aerobic glycolysis in EMT on cell motility, the effects of 2-DG and oligomycin treatment was assessed by time lapse microscopy. Treatment with 2-DG in the presence of pyruvate successfully prevented glycolysis-associated extracellular acidification without affecting OCR (Figures 5A and 5B). Conversely, OCR was reduced with concomitant increase in PPR upon addition of oligomycin (Figures 5C and 5D). After drug treatment, images were taken every 3 min for 6 hours (Time lapse images are available in Movie S1). The migration paths of PC3-Epi and PC3-EMT cells in the presence or absence of 2-DG or oligomycin were tracked every 15 min for 6 hours. Without any treatment, PC3-EMT cells migrated faster than PC3-Epi cells with respect to accumulated distance (Figures 5E and 5F). Treatment with 2-DG, but not oligomycin attenuated the motility of PC3-EMT and PC3-Epi (Figures 5E, 5F). Furthermore, MDA-EMT cells exhibited higher motility than MDA-Epi cells and treatement with 2-DG treatment, but not the oligomycin treatment, attenuated cell migration (Figure S4). These results suggest aerobic glycolysis, but not mitochondrial oxidative phosphorylation, is required for cell motility.

### Aerobic glycolysis is essential for cytoskeleton remodeling and the formation of focal adhesions

**Figure 6:**
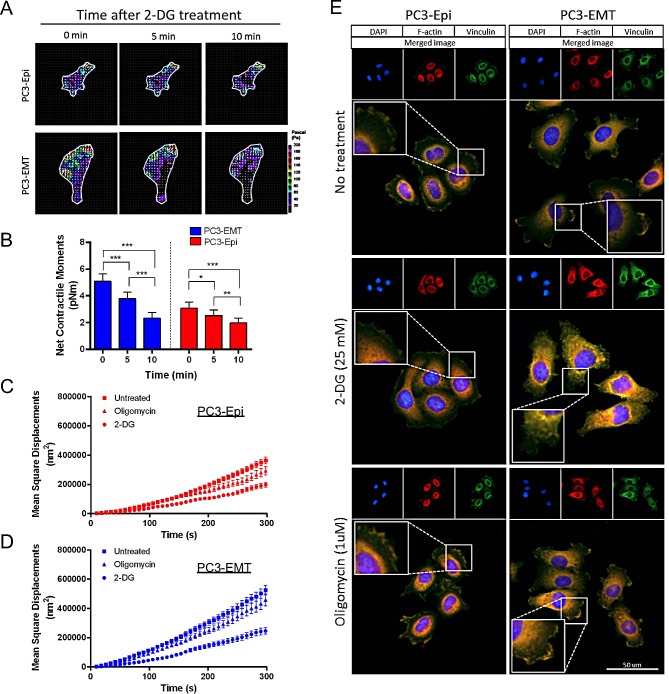
Aerobic glycolysis mediates the cytoskeleton remodeling and the formation of focal adhesions (A) Traction maps for representative PC3-Epi and PC3-EMT cells at baseline (0 min) in the absence of 2-DG, and at 5 min and 10 min in the presence of 2-DG (25 mM). The arrows overlaying each cell represent the direction and magnitude of the tractions, and the colors show the magnitude of tractions in Pa (see color scale). (B) Net contractile moments were obtained at all three time points. Data are represented as mean ± SE (*n* = 12 for PC3-EMT, *n* = 10 for PC3-Epi). Significance indicated by asterisks are p≤0.05 (*), p≤0.01 (**), and p≤0.001 (***). Remodeling represented by mean square displacements obtained from the spontaneous nanoscale bead motion in the presence or absence of 2-DG (25mM) and oligomycin in PC3-Epi (C) and PC3-EMT (D) cells. Data are represented as mean ± SE. (E) PC3-Epi and PC3-EMT cells were plated on type I collagen-coated slide and treated with 2-DG (25mM) or oligomycin (1uM) for 20 min. Cells were stained with DAPI for DNA (blue), phalloidin for polymerized actin (F-actin; red) and vinculin for focal adhesions (green).

In order to validate the association between aerobic glycolysis and cell motility, we measured cell traction forces and CSK remodeling in the presence of metabolic inhibitors. Individual PC3 cells showed some degree of heterogeneity in their responses to 2-DG (data not shown), however, all resulted in time-dependent decreases in cell traction forces (Figures 6A and 6B). PC3-EMT cells exhibited a greater sensitivity to the inhibition of glycolysis compared to PC3-Epi cells, showing the greatest reduction in net contractile moment with 2-DG (Figure 6B). In both PC3-Epi and PC3-EMT cells, CSK remodeling dynamics became markedly slower with 2-DG, but not with oligomycin (Figures 6C and D).

To confirm the effects of 2-DG and oligomycin on CSK remodeling and motility, the formation of focal adhesions was examined. PC3-Epi and PC3-EMT cells treated with 2-DG or oligomycin were stained with DAPI for DNA (blue), phalloidin for polymerized actin (F-actin; red) and vinculin for focal adhesions (green). The localization of focal adhesion formation in PC3-Epi and PC3-EMT cells exhibited different patterns: in PC3-EMT cells, focal adhesions were mainly localized in the lamellipodia of cells polarized toward the direction of cell movements (Figure 6E). PC3-Epi cells formed focal adhesions around the whole circumferential surface of the cell (Figure 6E). Importantly, the formation of focal adhesions was dramatically decreased by the inhibition of glycolysis after addition of 2-DG, but not by the inhibition of mitochondrial ATP synthesis with oligomycin (Figure 6E). The expression of proteins involved in the focal adhesions and the actin nucleation was examined by Western blot. No significant change was observed in vinculin, focal adhesion kinase (FAK), Diap1, and Diap2 expression levels with or without drug treatment (2-DG and oligomycin) in 20 minutes (Figure S5), implying the effects of drug treatment on CSK remodeling and motility were not due to changes in protein expression.

## DISCUSSION

In the present study, we investigated the role of metabolic alterations on cell motility and cytoskeleton remodeling in epithelial and mesenchymal cancer models. Our data reveal that increased glycolytic activity, but not mitochondrial respiration, imparts biophysical features distinguishing aggressive mesenchymal cancer cells from less-aggressive epithelial cancer cells. These glycolysis-dependent features include increased cell traction forces, cytoskeleton remodeling, and cell motility.

High dependency on ATP production on the glycolytic pathway (also known as the Warburg effect) is widely accepted as one of the most salient characteristics of cancer metabolism [31]. Our study demonstrates that aggressive mesenchymal cancer cells exhibit higher glycolytic activity which is associated with greater cell motility and faster CSK remodeling. Our findings are consistent with previous reports that metabolic shifts toward increased glycolytic activity are associated with a more aggressive phenotype [20]. Furthermore, the increased glycolytic activity observed in PC3-Epi cells as compared to non-cancerous prostate epithelial cell model suggests a fundamental metabolic transformation occurs in process of cancer formation.

A major finding of this study is that mesenchymal cancer cells acquire increased cell motility as compared to less aggressive epithelial cancer cells by increasing glycolytic activity without significantly altering mitochondrial ATP production. This is consistent with previous reports demonstrating that metabolic changes are associated with cellular differentiation and that the relative contribution of anaerobic metabolism and aerobic respiration may play an important role in determining specific lineage decisions [9-12]. Our results demonstrate that although epithelial and mesenchymal cancer cells exhibit a distinct phenotype with regard to cell motility, cell traction forces, and CSK remodeling, no significant difference in mitochondrial ATP production rate occurs. These findings suggest that metabolic alterations occur in epithelial and mesenchymal cancer cells to increase ATP production from glycolysis, but not from oxidative phosphorylation, a bioenergetic scheme consistent with a recent demand-driven model for glucose metabolism in which glycolysis and oxidative phosphorylation supply energy for different cellular processes [17].

In this report, we also show that inhibition of glycolysis attenuates cell motility even while mitochondrial ATP synthesis remains intact and that inhibition of mitochondrial respiration only minimally reduces cell motility compared to inhibition of glycolysis. These data indicate that in terms of energy utilization, ATP produced from oxidative phosphorylation is less readily used for cell migration compared to ATP generated from increased glycolysis. This is consistent with a recent report that hypoxic conditions increase cell migration [32] and is consistent with reports of glycolytic enzyme colocalization with sites of active cytoskeletal remodeling. Eukaryotic cells perform biochemical processes in a compartmentalized fashion, relying on various intracellular shuttles between organelles for increased control and regulation of physiological pathways. This suggests that providing the energy source for motility at the sites of CSK rearrangement (i.e., via glycolysis) is more favorable than exporting energetic molecules produced in the mitochondria to the cell periphery. Previous reports from outside the field of cancer have reported similar findings with respect to bioenergetics of cell motility: proliferating stalk endothelial cells and LPS-stimulated M1 polarized macrophages require glucose metabolism, but not mitochondrial oxidative phosphorylation, for control of lammelipodia formation and morphodynamics, respectively [33, 34]. These findings, taken with the data presented, suggest that glucose metabolism is a fundamental cellular process involved with general cellular motility and cytoskeletal rearrangement. Performing a ^13^C-glucose flux assay in the future could help confirm our findings.

Previous studies have documented that expression and activity of glycolytic enzymes including hexokinase and pyruvate kinase are deregulated in cancer cells [16]. Pyruvate kinase (PK) is an important regulator of the glycolytic pathway. Four isoforms of PK exist in mammals: PKL and PKR are transcribed from the PKLR gene with different promoters and PKM1 and PKM2 are encoded by the PKM gene and produced by alternative splicing [35, 36]. PKM2 is mainly expressed during embryogenesis and its expression is reduced concomitant with tissue-specific expression of PKL, PKR and PKM1. During tumorigenesis, the re-expression of PKM2 is observed in a variety of cancers including lung, breast, prostate, blood, cervix, kidney, bladder, and colon [37]. In our study, both PKM1 and PKM2 are up-regulated in mesenchymal cancer cells compared to epithelial cancer cells, resulting in high enzymatic activity of PK in mesenchymal cancer cells. It is important to note that PrECs exhibited the highest PKM activity among cell lines used in this study and the cell growth rate in mesenchymal cancer cells is less than the epithelial cancer cells (data not shown). This is consistent with a recent report that PKM2 is inactivated in proliferating tumor cells, whereas non-proliferating tumor cells require active pyruvate kinase [38]. Thus, re-activation of PKM2 might contribute to the metastatic process by increasing ATP generation from aerobic glycolysis.

There are four isoforms of hexokinase; HK1, HK2, HK3 and HK4 (also known as glucokinase). HK4 is distinct from other isoforms with respect to its restricted expression in the pancreas and liver and higher *K*m for glucose. HK3 is not functionally important since its activity is inhibited by physiological concentration of glucose [16]. It has been suggested that while HK1 is ubiquitously expressed in the majority of adult tissues, HK2 is expressed at high levels in many cancer cells [39, 40]. Several studies have demonstrated that HK2 plays a critical role in initiating and maintaining the high glucose catabolic rates of rapidly growing tumors [16, 39]. Our results show that while the expression of HK1 is up-regulated in PC3-EMT cells compared to PC3-Epi cells, no obvious difference is observed in HK2 expression between these cell lines. However, HK2 expression in PC3-Epi and PC3-EMT cells is significantly higher than in PrECs. Furthermore, the total activity of HK is up-regulated in PC3-EMT cells compared to PC3-Epi cells. These findings suggest the possibility that HK2 may play an important role in tumor initiation and development, but may not be directly involved in the metastatic process. Instead, up-regulation of HK1 expression followed by increased HK activity might be associated with aggressive cancer. On the other hand, HK activity is unchanged between MDA-Epi and MDA-EMT cells. MDA-Epi cells were established by the over-expression of OVOL1 and OVOL2, which is a different mechanism than used for the production of PC3-Epi and PC3-EMT cells. These results indicate that different mesenchymal cancer cell models may have different mechanisms to modulate glycolytic activity, further supporting the idea that increased reliance on the glycolytic may be a consequence of cancer progression and may be required for maintaining greater cell motility.

We have demonstrated that the biophysical properties of PC3-Epi and PC3-EMT cells differ both under resting conditions as well as in response to inhibition of aerobic glycolysis. Under resting conditions, PC3-EMT cells exhibit greater contractile strength and faster CSK remodeling dynamics than PC3-Epi cells. In addition, the inhibition of aerobic glycolysis through 2-DG treatment greatly suppresses the contractility of PC3-EMT cells while having a lesser effect on PC3-Epi cells. In terms of remodeling dynamics, CSK remodeling of PC3-EMT cells is greatly suppressed in the presence of 2-DG, while the suppressive effect of oligomycin on CSK remodeling is considerably less than with 2-DG. These results are consistent with the motility data demonstrating that the greater migratory ability of PC3-EMT cells is diminished by 2-DG treatment, but not oligomycin. Altogether, our results show that the two PC3 phenotypes exhibit biophysical differences with response to 2-DG, suggesting that aerobic glycolysis plays an important role in promoting the contractility and remodeling dynamics of PC3-EMT cells.

In summary, we provide new evidence that higher glycolytic activity in mesenchymal cancer cells is associated with greater cell motility and faster CSK remodeling. Mesenchymal cancer cells are energetically equivalent to epithelial cancer cells in terms of ATP production rate from mitochondria. Furthermore, the inhibition of glycolysis with 2-DG attenuates motility and CSK remodeling while mitochondrial respiration remains intact. However, inhibition of mitochondrial respiration while the glycolytic pathway remains intact only slightly affects motility and CSK remodeling. Taken together, these data suggest that ATP generated in the mitochondria is not readily rerouted to sites of active cytoskeleton remodeling upon glycolytic enzyme inhibition and that glucose metabolism plays a fundamental role in cytoskeletal remodeling and cellular motility.

## Methods

### Cell Culture

PC3-Epi and PC3-EMT cells were generated in our lab as previously described [29]. To generate PC3-Epi cells, colonies of PC3 cells were transfected with pLentilox-EV-Luc luciferase expression vector (University of Michigan Vector Core) and were selected based on an epithelial cell morphology, bioluminescent intensity and expression of E-cadherin. Selection was repeated twice and a stable epithelial population was obtained and further characterized by Western blot. PC3-EMT cells were isolated from PC3-Epi cells as previously described [29]. CD14+ monocytes were isolated from human peripheral blood mononuclear cells and were forced to differentiate into M2 macrophages upon stimulation with INFγ or interleukin (IL)-4 respectively (100 ng/ml) for 36-42 hours. The resulting M2 cells were co-cultured with 3×10^5^ highly epithelial prostate cancer cells (PC3-Epi) for 4 days. Cells were passaged 3 times through trypsinization and 10^4^ cells were plated onto 10 cm tissue culture plates. Populations showing change in morphology were further isolated and characterized by Western blot and gene expression analyses. Both cell lines were routinely maintained in RPMI 1640 with 10% fetal bovine serum (10%FBS) at 37°C in a humidified atmosphere containing 5% CO_2_. The normal human prostate epithelial cell line (PrEC) was obtained from Lonza (Walkersville, MD) and cultured in prostate epithelial cell medium (Lonza) at 37°C in a humidified atmosphere containing 5% CO_2_. Details can be found in Supplemental Experimental Procedures.

### Reagent and Antibody

Oligomycin A, carbonyl cyanide-4-(trifluoromethoxy)phenylhydrazone (FCCP), rotenone, antimycin A, and 2-deoxy-D-glucose (2-DG) were purchased from Sigma (St. Louis, MO). Anti-hexokinase 1 (HK1), anti-hexokinase 2 (HK2), anti-pyruvate kinase isoform M1 (PKM1), anti-pyruvate kinase isoform M2 (PKM2), anti-pyruvate dehydrogenase (PDH), anti-heat shock protein 60 (HSP60), superoxide dismutase 1 (SOD1), anti-cytochrome c (Cyt C), anti-succinate dehydrogenase complex, subunit A (SDHA), anti-cytochrome c oxidase IV (COX IV), anti-voltage-dependent anion channel (VDAC), prohibitins (PHB1), (Cell Signaling Technology, Inc., Danvers, MA), and anti-B-actin (Sigma) were used as the primary antibodies.

### Measurement of Oxygen Consumption Rate (OCR) and Proton Production Pate (PPR)

OCR and PPR were measured using a Seahorse Bioscience XF24 Extracellular Flux Analyzer (Seahorse Bioscience, North Billerica, MA) according to the manufacturer's protocol. Cells were seeded in normal growth medium 24 hours before measurement at a density of 2×10^4^ cells per well for PC3-Epi and PC3-EMT cells, 3×10^4^ per well for PrECs and 4×10^4^ per well for MDA-Epi and MDA-EMT cells. The assay was performed in XF minimal basal medium (Seahorse Bioscience) supplemented with 11mM glucose, 1mM sodium pyruvate and 1×GlutaMax (Life Technologies, Grand Island, NY) for OCR measurements and with 11mM glucose and 1×GlutaMax (Life Technologies) for PPR measurements. Cells were washed three times and pre-incubated in this medium for 1 hour before measurement at 37°C in a humidified atmosphere without CO_2_. Oligomycin A (1uM), FCCP (200nM for PC3-Epi and PC3-EMT, and 400nM for PrECs), rotenone (2uM), antimycin A (2uM), and 2-DG (100mM) were used to evaluate mitochondrial respiratory capacity and glycolytic capacity. The data were normalized by the amount of protein present in each well. We confirmed that normalization to the number of cells by measuring DNA content was correlated with the normalization ratio as measured by total amount of protein.

### DNA extraction and mitochondrial DNA (mtDNA) copy number measurement

Total DNA was extracted from cells using a DNeasy Tissue Kit (Qiagen, Valencia, CA). The quantitative real-time PCR (Q-PCR) was performed with 1μL (10ng) of DNA template in 25 μL of reaction mixture containing 12.5 μL of iQ SYBR Green Supermix and 0.25 μmol/L each primer. PCR reactions were subjected to hot start at 95°C for 3 minutes followed by 45 cycles of denaturation at 95°C for 10 seconds, annealing at 60°C for 30 seconds, and extension at 72°C for 30 seconds using the CFX96 Real-Time PCR Detection System (Bio-Rad Laboratories, Inc., Hercules, CA). The sequence of PCR primers used in this study was described in Table S1. The ratio of mtDNA to nuclear DNA was calculated by dividing copies of ND1 or ND5 with copies of SLCO2B1 and SERPINA1.

### RNA extraction and quantitative real-time PCR

Total RNA was isolated using the RNeasy Kit (Qiagen). First strand cDNA was made from 1 μg RNA using iScript cDNA Synthesis Kit (Bio-Rad Laboratories, Inc.) following the manufacturer's protocol in a total volume of 20 μL. Quantitative real-time PCR (Q-PCR) were carried out as mtDNA copy number measurement. The sequence of PCR primers used in this study was described in Table S1. TATA binding protein (TBP) was used as an internal control. Analysis and fold differences were determined using the comparative threshold cycle method.

### Western blot

Cell lysates were prepared in whole cell lysis buffer (50 mM Tris-HCL pH 7.5, 1% SDS) with 1 mM dithiothreitol, 1 mM phenylmethylsulfonyl fluoride and 1 × Halt Protease and Phosphatase Inhibitor Cocktail (Thermo Fisher Scientific, Rockford, IL) followed by sonication and centrifugation. Extracts were quantified using the Bio-Rad Protein Assay (Bio-Rad Laboratories, Inc.). Lysates were subjected to SDS–PAGE, and transferred to nitrocellulose membranes (Bio-Rad Laboratories, Inc.). Membranes were incubated with primary antibodies followed by a secondary IRDye IgG antibody (Li-Cor Biosciences, Lincoln, NE). Images were detected by the Odyssey Infrared Imaging System (Li-Cor Biosciences).

### Enzyme activity assay

Hexokinase and pyruvate kinase activity was measured by Hexokinase Colorimetric Assay Kit and Pyruvate kinase Assay Kit (Abcam, Cambridge, MA) following manufacturer's protocol. Samples were incubated with reaction mix and background control mix for 60 minutes at room temperature. Absorbance was measured at 450 nm for hexokinase and 570 nm for pyruvate kinase with FLUOstar Omega microplate reader (BMG LABTECH, Ortenberg, Germany). The absorbance from all sample readings was corrected by subtracting each value derived from the background control and normalized by the amount of protein in the sample. The signals taken from two time points in linear range were used to calculate enzymatic activity. Hexokinase activity was determined by the glucose-6-phosphate dependent conversion of NAD+ to NADH, and 1.0 unit of hexokinase is defined as the amount of enzyme that will generate 1.0 umol of NADH per min at pH 8 at room temperature. Pyruvate kinase activity was determined by the pyruvate generation and 1.0 unit of pyruvate kinase is defined as the amount of enzyme that will transfer a phosphate group from PEP to ADP, yielding 1.0 umol of pyruvate per min at room temperature.

### Immunofluorescence

Cells were plated on collagen-I coated 4-chamber cell culture slide (BD Biosciences, Bedford, MA) 48 hours before fixation. Then, cells were fixed with 4% methanol-free formaldehyde (Thermo Scientific) for 15 min, permeabilized with 0.1% Triton X-100 for 10 min and blocked with image-iT FX Signal Enhancer (Life Technologies) for 30 min. Vinculin antibody (Sigma) was used to stain focal adhesion formations and actin filaments were stained by Alexa-Fluor 546-conjugated phalloidin (Invitrogen). ProLong® Gold Antifade Reagent with DAPI (Life Technologies) was used as a mountant. Images were taken by EVOS FL Auto microscope (Life Technologies).

### Fourier Transform Traction Microscopy

Traction forces were measured with Fourier transform traction microscopy as described previously [41-43]. In brief, cells were plated sparsely on collagen-coated elastic gel blocks (Young's modulus of 8,000 Pa and Poisson's ratio of 0.48) and allowed to adhere and stabilize for 24 hours in RPMI 1640 medium at 37°C in humidified air containing 5% CO_2_. Unless otherwise stated, adherent cells were extensively washed in XF minimal basal medium supplemented with 11 mM glucose, 1 mM sodium pyruvate, and 1× GlutaMax (Invitrogen). For each adherent cell, images of fluorescent microbeads (0.2 μm in diameter, Molecular Probes, Eugene, OR) embedded near the gel apical surface were taken at different times (i.e., before, and 5 and 10 min after 25 mM 2-DG); the fluorescent image of the same region of the gel after cell detachment with trypsin was used as the reference (traction-free) image. The displacement field between a pair of images was then obtained by identifying the coordinates of the peak of the cross-correlation function [43]. From the displacement field and known elastic properties of the gel, the traction field was computed using both constrained and unconstrained Fourier transform traction cytometry [43]. The computed traction field was used to obtain net contractile moment, which is a scalar measure of the cell's contractile strength and is expressed in units of pico-Newton meters (pNm).

### Spontaneous Bead Motions with Magnetic Twisting Cytometry with Optical Detection

Dynamic changes in CSK remodeling were measured by spontaneous motions of RGD-coated ferromagnetic microbeads (4.5 μm in diameter) bound to the CSK through cell surface integrin receptors as previously described [44, 45]. For this study, cells were plated at 200,000 cells/cm^2^ on plastic wells (96-well Removawell, Immulon II: Dynetech) coated with collagen type I at 500 ng/cm^2^. Cells were then incubated for the indicated time, in the presence or absence of 2-DG (25 mM) and oligomycin (1uM).

### Microscopic examination

Phase contrast images of cells were taken every 3 minutes for 6 hours after 2-DG (25mM) and oligomycin (1uM) treatment using EVOS FL Auto microscope (Life Technologies). Accumulated distance was analyzed using ImageJ software (http://imagej.nih.gov/ij/index.html) with Manual Tracking (http://rsb.info.nih.gov/ij/plugins/track/track.html) and with the Chemotaxis and Migration Tool Version 1.01 (http://ibidi.com/software/chemotaxis_and_migration_tool/).

### Statistical Analysis

Statistical differences between two groups of data were analyzed using the Student's t test. The Tukey-Kramer test was employed to analyze statistical differences among more than three groups of data. Statistical significance was applied to *P* values of less than 0.05.

## SUPPLEMENTARY MATERIAL, TABLES AND FIGURES


