# Supplementary material for: Glycolysis is the primary bioenergetic pathway for cell motility and cytoskeletal remodeling in human prostate and breast cancer cells
Source: Oncotarget. 2014 Nov 16;6(1):130–43. doi: 10.18632/oncotarget.2766 (PMC4381583; doi:10.18632/oncotarget.2766)
Supplement: Supplementary file 1 [file oncotarget-06-130-s001.pdf]

Glycolysis is the primary bioenergetic pathway for cell motility and cytoskeletal remodeling in human prostate and breast cancer cells

Supplemental Material

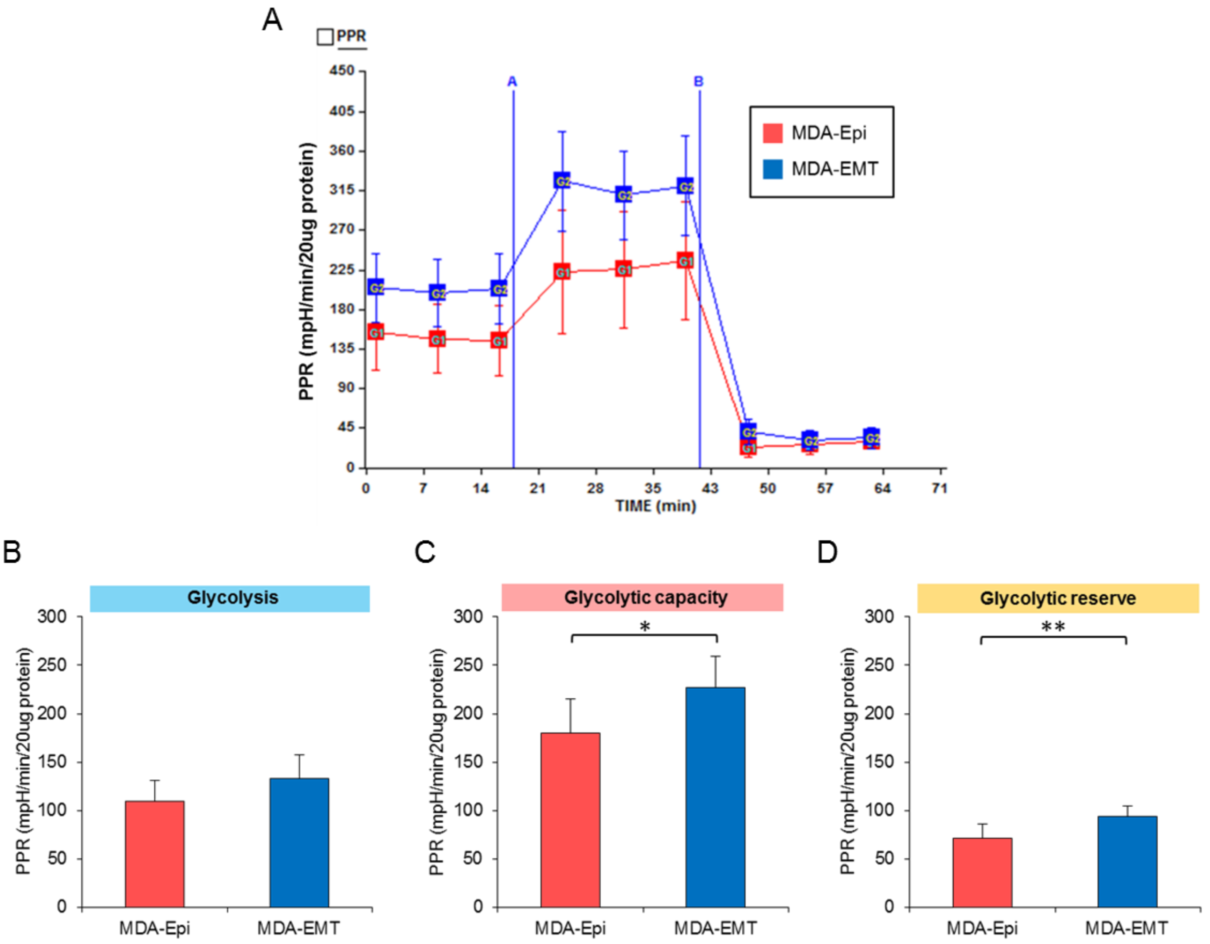

Figure S1: MDA-EMT cells have higher glycolytic activity compared to MDA-Epi cells, related to Figure 1.

The poorly differentiated (mesenchymal-type) MDA-MB-231 cells (denoted MDA-EMT) were obtained from ATCC. OVOL1 and OVOL2 over-expressing (epithelial-type) MDA-MB-231 cells (denoted MDA-Epi) were established as previously described (Roca et al., 2013). Their distinct epithelial and mesenchymal phenotype were confirmed by Western blot for epithelial and mesenchymal marker including E-cadherin, ZEB1, ZEB2, snail and slug (Roca et al., 2013). (A)

Representative traces of PPR in MDA-Epi and MDA-EMT cells. PPR was measured continuously throughout the experimental period at baseline followed by the addition of the indicated drugs. A; oligomycin (1uM), B; 2-DG (100mM). Glycolysis (B), glycolytic capacity (C) and glycolytic reserve (D) were calculated from the mean of three baseline readings. The independent biological experiments were repeated at least three times. Data were represented as the mean ± SD from 8 or 10 Seahorse microplate wells. *$P<0.05$, **$P<0.01$.

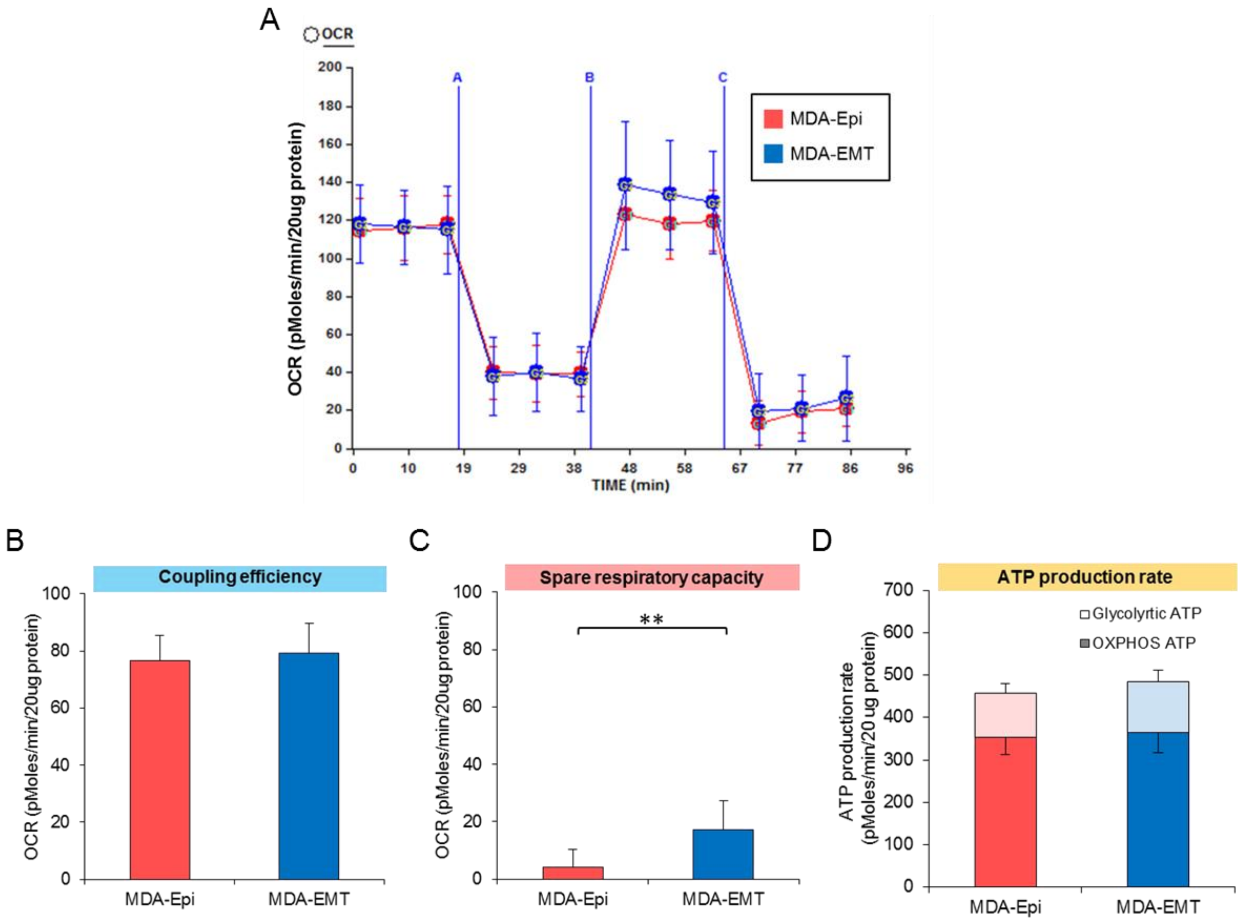

Figure S2: MDA-Epi and MDA-EMT cells exhibit a similar mitochondrial ATP production rate, related to Figure 3.

(A) Representative traces of OCR in MDA-Epi and MDA-EMT cells. OCR was measured continuously throughout the experimental period at baseline followed by the addition of the indicated drugs. A; oligomycin (1uM), B; FCCP (400nM), C; Antimycin A (2uM) and Rotenone (2uM). Coupling efficiency (B) and spare respiratory capacity (C) were calculated from the mean of three baseline readings. (D) ATP production rate was calculated from OCR and PPR measured in the Seahorse Bioscience XF24 Extracellular Flux Analyzer by the following equation; ATP production rate = OCR x 4.6 + PPR x 1. The independent biological experiments were repeated at

least three times. Data were represented as the mean ± SD from 8 or 10 Seahorse microplate wells. *$P<0.05$.

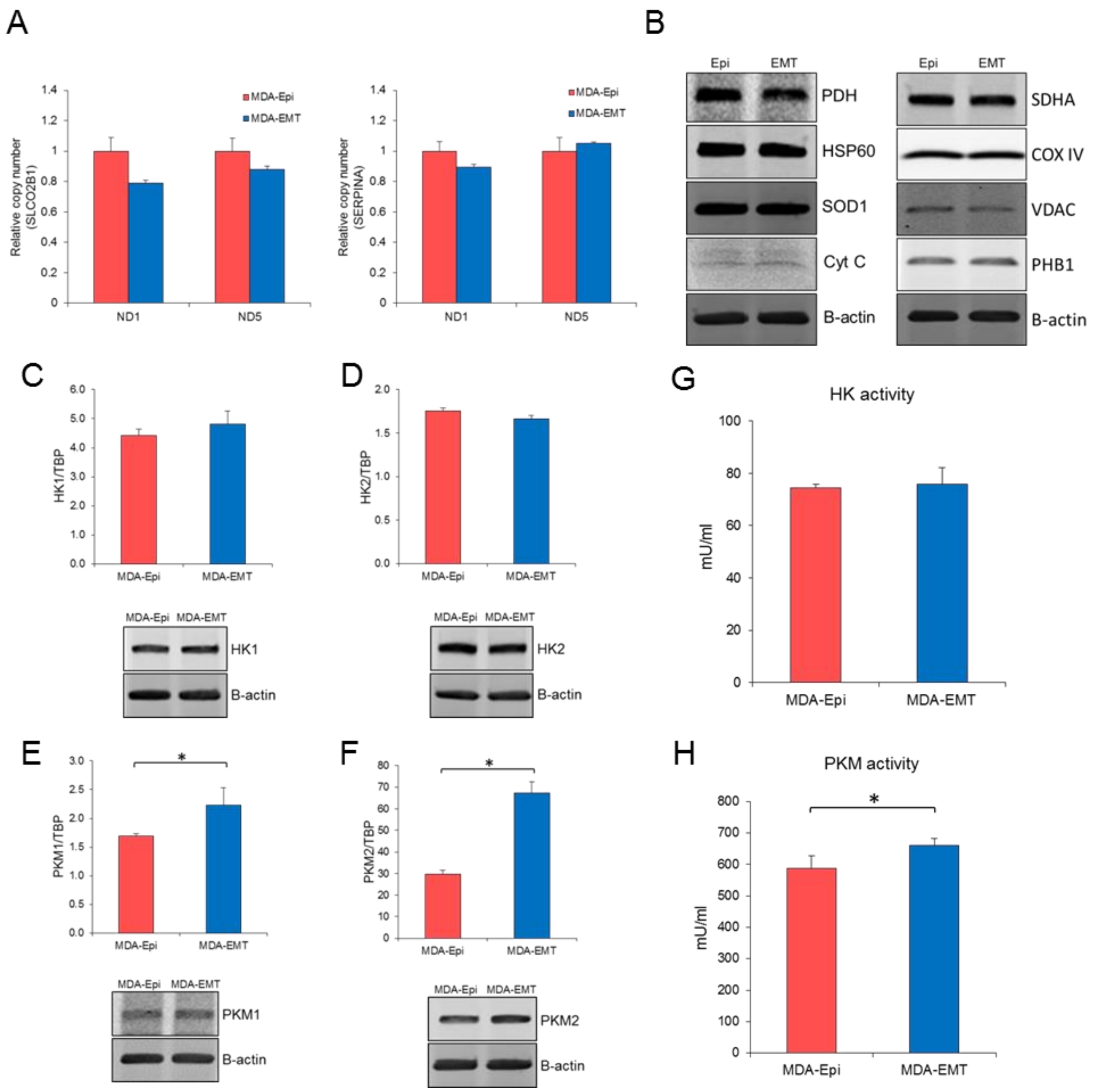

Figure S3: Higher expression and activity of glycolytic enzyme in MDA-EMT cells, related to Figure 4.

The ratio of NADH dehydrogenase subunit 1 (ND1) and NADH dehydrogenase subunit 5 (ND5) to SLCO2B1 (A) and SERPINA1 (B) DNA in MDA-Epi and MDA-EMT cells. The amount of ND1, ND5, SLCO2B1 and SERPINA1 were analyzed by Q-PCR. Data were represented as the mean ± SD from three independent experiments. (C) Mitochondrial proteins were detected by

western blots in MDA-Epi and MDA-EMT cells. B-actin was used as a loading control. HK1 (C), HK2 (D), PKM1 (E) and PKM2 (F) mRNA expression (upper) were determined in MDA-Epi and MDA-EMT cells by Q-PCR. The expression of mRNA was normalized to TATA binding protein (TBP). Data were represented as the mean ± SD from three independent experiments. Western blots for HK1 (C), HK2 (D), PKM1 (E) and PKM2 (F) protein expression (lower) in PrEC, PC3-Epi and PC3-EMT cells. B-actin was served as a loading control. Hexokinase (G) and pyruvate kinase (H) activities in MDA-Epi and MDA-EMT cells. The independent biological experiments were repeated at least three times. Data were represented as the mean of triplicate experiments ± SD. *P<0.05.

Movie S1: Inhibition of glycolysis, but not mitochondrial ATP synthesis, attenuates cell motility in PC3-Epi and PC3-EMT cells, related to Figure 5.

Provided as multimedia files (.avi).

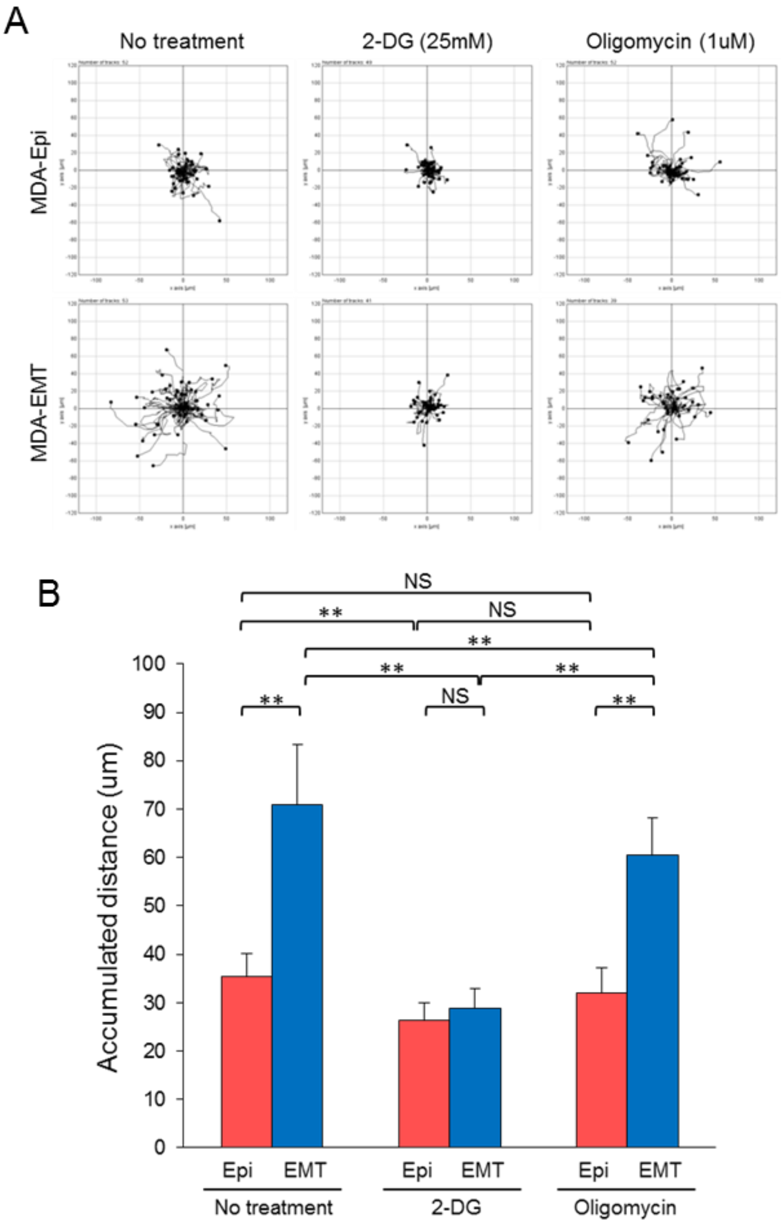

Figure S4: Inhibition of glycolysis, but not mitochondrial ATP synthesis, attenuates cell motility in epithelial and mesenchymal cancer cell models derived from breast cancer cell lines, related to Figure 5.

MDA-Epi and MDA-EMT cells were cultured in XF minimal basal medium supplemented with 11mM glucose, 1mM sodium pyruvate and 1xGlutaMax in the presence or absence of 2-DG (25mM) or oligomycin (1uM). (A) Representative tracks of cell movements that were traced and visualized using Manual Tracking and Chemotaxis tool of ImageJ software every 15 min for 6

hrs. (n=52 and 53 for no treatment, n=49 and 41 for 2-DG treatment and n=52 and 39 for oligomycin treatment in MDA-Epi and MDA-EMT cells, respectively.) (B) The accumulated distance analyzed by chemotaxis tool of ImageJ software. Data are represented as mean of triplicate experiments ± SD. **P<0.01. NS; not significant.

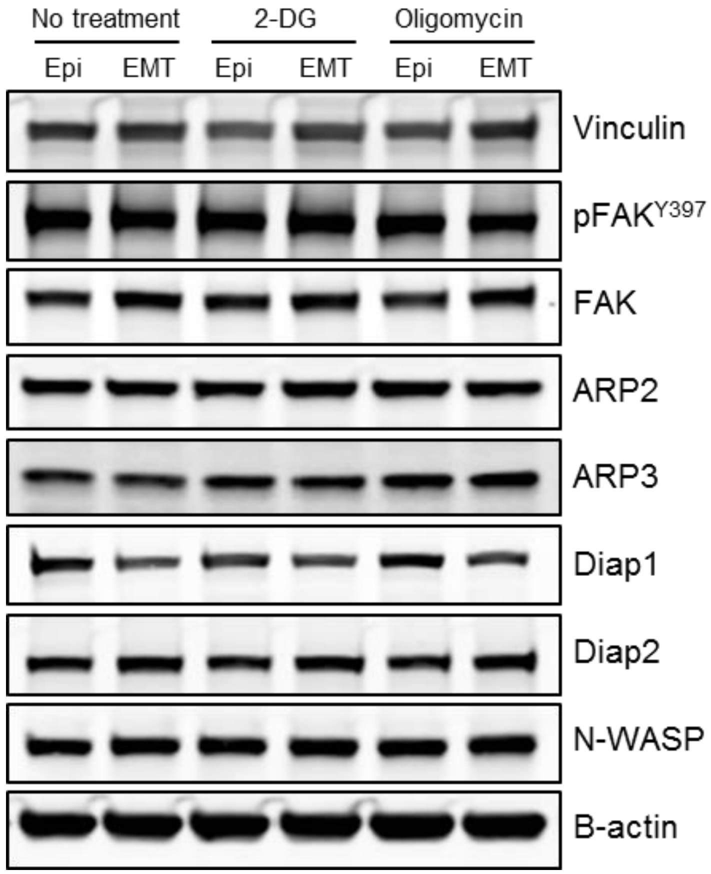

Figure S5: Inhibition of glycolysis did not affect the expression of proteins involved in the focal adhesions and the actin nucleation, related to Figure 6.

PC3-Epi and PC3-EMT cells were cultured in XF minimal basal medium supplemented with 11mM glucose, 1mM sodium pyruvate and 1xGlutaMax in the presence or absence of 2-DG (25mM) or oligomycin (1uM) for 20min. The expression of proteins involved in the focal adhesions and the actin nucleation was analyzed by Western blots. Anti-actin-related protein 2 (ARP2), anti-actin-related protein 3 (ARP3), anti-diaphanous-related formin 1 (Diap1), anti-diaphanous-related formin 2 (Diap2), anti-Wiskott-Aldrich syndrome-like (N-WASP) (Cell Signaling Technology, Inc.), anti-focal adhesion kinase (FAK), anti-FAK (phospho Y397) (Abcam), anti-vinculin (Sigma) were used as the primary antibodies. B-actin was served as a loading control.

Table S1: Primer sequences for quantitative real-time PCR, related to Experimental Procedures.

Gene	Forward primer sequence	Reverse primer sequence
**HK1**	GGACTGGACCGTCTGAATGT	ACAGTTCCTTCACCGTCTGG
**HK2**	CAAAGTGACAGTGGGTGTGG	GCCAGGTCCTTCACTGTCTC
**PKM1**	CTATCCTCTGGAGGCTGTGC	CCATGAGGTCTGTGGAGTGA
**PKM2**	CCACTTGCAATTATTTGAGGAA	GTGAGCAGACCTGCCAGACT
**ND1**	CCTAAAACCCGCCACATCTA	GCCTAGGTTGAGGTTGACCA
**ND5**	TCCTCGCCTTAGCATGATTT	TGAGGCTTGGATTAGCGTTT
**SLCO2B1**	GCACTGCATCACTGGCTTTA	TTACTTTGGGGTGAGCTTGG
**SERPINA1**	ATGCTGCCCAGAAGACAGAT	ATAGGCTGAAGGCGAACTCA
**TBP**	GAATATAATCCCAAGCGGTTTG	ACTTCACATCACAGCTCCCC
